# The adrenal-vitamin C axis: from fish to guinea pigs and primates

**DOI:** 10.1186/s13054-019-2332-x

**Published:** 2019-01-28

**Authors:** Michael H. Hooper, Anitra Carr, Paul E. Marik

**Affiliations:** 1 Division of Pulmonary and Critical Care Medicine, Eastern Virginia Medical School, 825 Fairfax Av, Suite 410, Norfolk, VA 23507 USA; 2 Department of Pathology, University of Otago, Christchurch, PO Box 4345, Christchurch, 8140 New Zealand

**Keywords:** Vitamin C, Cortisol, Stress response

Primates and guinea pigs are unable to synthesize vitamin C. In contrast, almost all other mammals produce vitamin C in their livers with production increasing during stress. Furthermore, largely to metabolic consumption, a high percentage of critically ill patients are deficient in vitamin C. In an observational study, Carr et al. found that 75% of critically ill patients had plasma levels of vitamin C that were abnormally low [1]. The degree and incidence of deficiency were most pronounced in those patients with sepsis. Several trials have shown that administration of vitamin C to patients with sepsis is associated with better patient outcomes, suggesting a causal relationship between vitamin C deficiency and outcome. The mechanism(s) by which vitamin C administration may improve outcomes is unclear. Observations of very high vitamin C levels in the adrenal gland as well as its release in response to ACTH suggest that vitamin C plays a role in the stress response [2]. Release of cortisol in response to stress is well documented in humans and throughout the animal kingdom. However, there is marked inter-species variation in the amount of cortisol released in response to a stressor. Interestingly, there is a strong inverse correlation between the ability of an animal to endogenously produce vitamin C and the cortisol response when stressed. Barton et al. reported the baseline cortisol and response of numerous fish species to handling [3]. Those fish species which synthesized vitamin C had a 1.6-fold increase in cortisol levels after stress as compared to a 20.2-fold increase in those fish species that were unable to produce vitamin C, with the non-producers having a significantly higher baseline cortisol level. Additional evidence supports the concept of an inverse correlation between vitamin C and cortisol levels. Guinea pigs that are made deficient in vitamin C hyper-secrete cortisol [4]. Supplementation of ascorbic acid in humans and animal models is associated with a decreased cortisol response after a psychological or physical stressor [5]. High serum levels of cortisol in patients with sepsis are associated with a poor prognosis. Traditionally, this association has been explained on the assumption that higher cortisol responses are due to a more intense physiological stress and a higher severity of illness. However, the inverse relationship of cortisol levels with vitamin C status would suggest an alternative hypothesis, namely, that high levels of cortisol and the associated poorer outcomes of patients are a function of vitamin C deficiency.
